# Selective Ion Separation by Capacitive Deionization: A Comprehensive Review

**DOI:** 10.3390/ma18051107

**Published:** 2025-02-28

**Authors:** Fanyi Xu, Ling Yuan, Rui Zhao, Bing Qin, Feng Zhang, Liming Ren, Hailun Yang, Menglei Yuan

**Affiliations:** 1 Sinopec Research Institute of Petroleum Processing Co., Ltd., Beijing 100083, China; xufanyi.ripp@sinopec.com (F.X.); zhaorui.ripp@sinopec.com (R.Z.); qinbing.ripp@sinopec.com (B.Q.); zhangfeng.ripp@sinopec.com (F.Z.); renliming.ripp@sinopec.com (L.R.); 2 State Key Laboratory of Pollution Control and Resource Reuse, School of the Environment, Nanjing University, Nanjing 210023, China; yuanling@ipe.ac.cn; 3 State Key Laboratory of Solidification Processing, School of Materials Science and Engineering, Queen Mary University of London Engineering School, Northwestern Polytechnical University, Xi’an 710072, China

**Keywords:** capacitive deionization, ion separation, desorption, electrode materials

## Abstract

Within the last decade, in addition to water desalination, capacitive deionization (CDI) has been used for the resource recovery and selective separation of target ions in multicomponent solutions. CDI is a new technology for selectively extracting valuable metal ions from solutions using an electric field and electrode materials. Unlike traditional adsorption methods, it raises attention for its environmentally friendly process and low cost, especially for extracting valuable elements. CDI technology has advanced significantly in desalination and selective element extraction due to a deep understanding of ion storage, electrode material structure–activity relationships, solvent effects, and reactor design. However, it still faces challenges like short electrode cycle life, poor reversible absorption/desorption, low charge utilization, and limited ion selectivity. In this review, we commence with an examination of the historical development of CDI technology, followed by a comprehensive summary of the fundamental operating principles of capacitors. We then evaluate the criteria for assessing capacitor performance and analyze the advantages and disadvantages associated with various capacitor materials. According to the review, we address the current challenges and obstacles encountered in the advancement of capacitor technology and offer constructive recommendations for its future development.

## 1. Introduction

**Figure 1:**
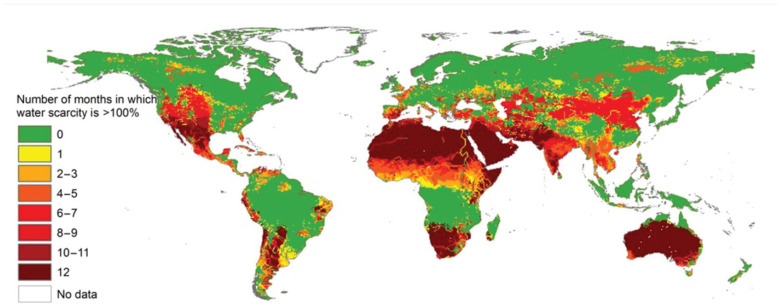
The blue water scarcity in months per year from 1996 to 2005. Reproduced under the terms of the CC BY-NC license. Copyright 2016, the authors, published by the American Association for the Advancement of Science.

Water is essential for life, but global consumption has made it scarce. Figure 1 demonstrates the blue water deficit in months during each year between 1996 and 2005. Considering sustainable development, recycling fresh water and extracting valuable elements are now crucial in environmental science [1]. Traditional wastewater treatment methods, which are destructive, no longer meet modern needs for resource recycling. This realistic challenge has prompted the urgent need for technological innovation in wastewater treatment and has strongly promoted the innovation and upgrading of water treatment technology [2].

Capacitive deionization (CDI), first conceptualized in 1960 by Blair and Murphy and later termed “capacitive deionization” by Farmer et al. in 1995, is gaining attention as a competitive desalination method [3]. It is particularly suitable for applications with salt concentrations below 10 g/L, such as household or small business use, due to its low pressure and temperature requirements and the lack of need for trained operators. The system runs on low voltages (≈1 V) similar to common consumer electronics, allowing for unsupervised operation. Consequently, CDI has become a promising desalination technology in recent decades. Concurrently, significantly increasing attention is being directed toward CDI for ion exchange and recovery applications, largely due to the extensive and expanding array of modifiable capacitive materials available [4].

Various strategies have been proposed to enhance or introduce selectivity in pristine CDI electrodes. These strategies encompass the utilization of electrode materials with diverse pore sizes and compositions [5], the incorporation of functional groups, the application of standard or specialized ion-selective membranes, and the optimization of operational parameters, either individually or in combination [6]. In recent years, research on CDI has been dedicated to advancing electrode materials and exploring theoretical principles to drive innovation [7]. However, there is a lack of comprehensive articles that systematically integrate these aspects. Therefore, this review aims to discuss the principles underlying the ion extraction mechanism of CDI, the synthesis of electrode materials, and the methodologies employed in its study, utilizing advanced characterization techniques and theoretical tools.

## 2. Desalination Method

Desalination is a process used to remove dissolved salts from saltwater, creating fresh water. Depending on the primary driving forces behind the process, desalination can be categorized into three main types, namely pressure-driven, thermal, or electrokinetic. These methods aim to reduce the salt concentration in saline media while preserving other beneficial components of the water.

### 2.1. Pressure-Driven Desalination

Hydraulic pressure gradients are utilized in pressure-driven desalination systems to partition water molecules through a membrane, ensuring the removal of dissolved salts while preserving beneficial components in the feed stream. This method typically operates under specific conditions, such as a high salt concentration in the feed and a narrow temperature range, to efficiently desalinate water. Membrane-based water treatment technologies can be categorized into reverse osmosis, nanofiltration, ultrafiltration, and microfiltration based on the membrane pore size [8]. Reverse osmosis utilizes a selectively permeable material with pores ranging from 0.1 to one nanometer to purify water and eliminate impurities. This process produces two streams, namely a desalinated water stream and a concentrated brine stream. For reverse osmosis to function effectively, there must be sufficient pressure applied to overcome the osmotic pressure differential between feedwater and permeate. Developed in the 1950s, reverse osmosis is now a well-established technology widely used for seawater and brackish water desalination. While it is highly effective at removing small-sized salts, membrane fouling and contamination remain significant challenges that impact its long-term performance [9]. Membrane separation technologies such as nanofiltration, ultrafiltration, and microfiltration employ membranes with pore sizes of 1–10 nm, 10–100 nm, and 100 nm–10 μm, respectively. When hydraulic pressure is introduced, these systems efficiently block the passage of divalent ions, sub-molecular organic compounds, proteins, macromolecules, viruses, and other contaminants from the feedwater, ensuring a cleaner filtration process.

### 2.2. Thermal Desalination

Thermal desalination involves the separation of water from nonvolatile solutes via a process of evaporation and subsequent condensation. Among the earliest desalination techniques, distillation operates on the principle that a significant amount of energy is required to overcome the latent heat of vaporization of saline water, typically around 677 kWh m^−3^ [10]. Popular thermal desalination techniques involve processes such as multi-stage flash desalination systems (MSF) and multi-step evaporation processes (MEE), which are widely used for efficient water treatment [11]. The workflow of multi-stage flash desalination is as follows: initially, feedwater is heated to a temperature just below its boiling point. The heated feedwater is then transferred to the next stage, where it vaporizes due to a continuous reduction in pressure. Throughout this operation, the feed water’s ionic components accumulate in a concentrated brine solution at the lower part of the initial stage. The initial stage’s vaporized water serves to elevate the temperature and vaporize new feedwater as it enters the second stage, resulting in further ion separation and the production of additional ion-free steam. The vapor is then cooled and transformed into freshwater. The resulting freshwater and concentrated saltwater from the initial phase are directed to the next phase at reduced pressure, and this cycle continues through several phases until all the condensed freshwater is accumulated.

In multiple-effect evaporation desalination, the process involves multiple stages or “effects”. Feedwater is heated in tubes using steam in each effect, causing part of the water to evaporate. The resulting steam then enters the tubes of the next effect, generating more distilled vapor, which condenses into freshwater. Both MSF and MEE are energy-intensive and complex processes, requiring significant power consumption and multiple stages to achieve efficient desalination.

### 2.3. Electrokinetic Desalination

Electrokinetic desalination utilizes electrical voltage or current to facilitate the movement of charged ions. Electrokinetic desalination primarily consists of two methods, namely electrodialysis and capacitive deionization (CDI). Electrodialysis, a well-established technique, involves the arrangement of cation-exchange and anion-exchange membranes in alternating patterns between channels. In this process, anodes and cathodes generate an electrical force that propels cations and anions through these membranes. This movement transfers ions from one solution to another, resulting in the production of both concentrated and diluted streams [12]. However, electrodialysis necessitates comparatively high voltages, generally above 20 V, to facilitate ion movement, accomplishing the separation of ions but simultaneously leading to the decomposition of water [13].

CDI is a developing technique designed for the efficient elimination of charged ions from water solutions [14]. In recent years, there has been significant progress in CDI development, encompassing mathematical model advancements, novel electrode material innovations, cell structure designs, and additional improvements. A conventional CDI assembly is characterized by two porous carbon electrodes, partitioned by an insulating medium through which the influent water permeates for treatment.

Under a voltage differential ranging from 0.8 to 2.0 volts, the electrodes induce the migration of salt ions in the feedwater. This migration occurs toward the electrical double layers (EDLs) formed along the pore surfaces at the electrode–water interface. This process, referred to as electrosorption, facilitates the separation of salts from the feedwater [15]. Upon discontinuation of the external power supply or reversal of its polarity, the ions adsorbed within the system are desorbed. This discharge process leads to the liberation of ions, resulting in a concentrated brine stream. Additionally, the electrical charge exiting the cell during this phase can be harnessed for energy recovery purposes.

CDI offers several advantages over other desalination methods. Firstly, it is a straightforward and simple process, relying on electro-adsorption, which eliminates the need for extreme conditions like high pressure or high temperature. It can be powered by renewable energy sources, such as solar or wind power, as well as rechargeable batteries. This makes it an ideal on-site desalination solution. Such solutions are particularly beneficial in remote areas, where access to high-energy power is limited. Additionally, CDI is environmentally friendly, as it does not require the use of chemicals and does not produce secondary pollutants. Furthermore, CDI represents an energy-efficient technology distinguished by its high capacity for energy recovery. This is primarily due to its mechanism of ion extraction from water instead of employing a water–salt separation process. Operating on principles akin to those of a capacitor, CDI facilitates substantial energy recovery during the discharge phase. It is also characterized by a rapid rate of desalination and exhibits long-term cycling stability, further enhancing its sustainability and efficiency in various applications.

## 3. CDI Evolution and Working Principles

### 3.1. Evolution of CDI

**Figure 2:**
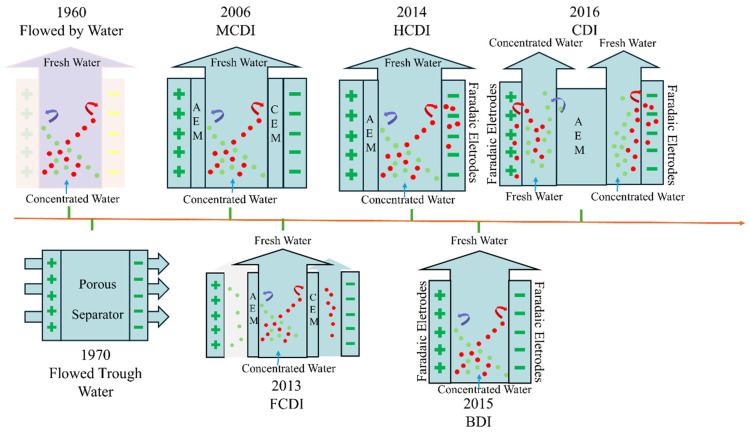
CDI system configuration development diagram. (AEM: anion-exchange membrane; CEM: cation-exchange membrane).

Scientists started studying CDI technology in the 1960s, with Blair and Murphy introducing the concept and calling the first CDI structure “Flow-by capacitive deionization [16]”. In 1970, Johnson and colleagues discovered that ion adsorption followed the double-electric-layer theory through their research on desalination using porous carbon electrodes. They devised a flow-through capacitive deionization (FCDI) system. In this system, the liquid stream is conveyed directly through the electrodes [17]. This innovation led to the evolution of today’s common CDI devices into plate and coil designs. In 2006, Lee et al. introduced ion-exchange membranes (IEMs) to CDI, creating membrane capacitive deionization (MCDI) [18]. In 2010, Biesheuvel et al. further explained MCDI’s theory and electrode pore effects [19]. MCDI improves upon conventional CDI through the incorporation of anion-exchange and cation-exchange membranes at the anode and cathode, respectively. After the voltage is applied, the cation is transported to the cathode through the cation-exchange membrane, and the anion is transported to the anode through the anion-exchange membrane, thus achieving desalination [20]. However, MCDI faces increased resistance due to internal, interfacial, and contact resistances. Wu et al. enhanced the issue by integrating a membrane with the carbon electrode, avoiding a separate IEM [21]. This integrated MCDI improved counterion selectivity, reduced electrical resistance, increased adsorption rate, and lowered energy consumption. However, more research is needed on the Faraday reaction in integrated MCDI, particularly regarding the IEM’s impact on capacitance and the Faraday current. In 2013, Jeon introduced flow capacitive deionization (FCDI), which replaces the fixed electrodes in traditional CDI with a flowing electrode slurry [22]. FCDI operates similarly to CDI, applying a constant voltage or current to facilitate ion migration through an ion-exchange membrane. The flow electrode allows for adsorption onto suspended carbon material, but its drawbacks include discontinuous conductivity, low electron transfer efficiency, and limited ion adsorption capacity. The high-energy-consumption characteristics, to a certain extent, limit the widespread application of FCDI technology in the industrial field [23]. To address low desalination efficiency, Lee et al. introduced hybrid capacitive deionization (HCDI) in 2014, enhancing CDI technology for better performance [24]. The design combines the features of standard CDI and battery systems, preserving CDI’s rapid desalination while boosting performance and efficiency in water treatment. As an asymmetric system, HCDI employs a sodium–manganese oxide electrode. It also utilizes an anion-exchange membrane and a porous carbon electrode. This combination enables a distinctive desalination mechanism. In desalination, sodium–manganese oxide electrodes capture sodium ions, and chloride ions are retained on porous carbon electrodes. Key HCDI materials include sodium–manganese oxides, Prussian blue derivatives, and NASICON-type materials [7]. While cathode-focused HCDI systems are well studied, anode-focused HCDI research is limited [25]. A study developed a novel A-HCDI system using PTMA as a selective anion adsorption electrode paired with a commercial AC cathode, significantly improving long-term stability and reducing energy loss from side reactions compared to membrane-free CDI. Recently, Liu et al. also created a new asymmetric capacitive deionization system (A-CDI) based on differing electromigration rates [26]. The positive and negative electrodes have different active material mass loadings. Electrosorption experiments revealed that A-CDI (asymmetric capacitive deionization) achieved higher desalination efficiency and faster adsorption rates than traditional symmetric CDI electrodes. This finding advances the utilization of electrode materials and provides a novel methodology for the development of high-performance CDI devices. Increasing the mass of active materials on the negative electrode reduces its efficiency. Thus, finding the optimal ratio of active materials between both electrodes to maximize their synergistic effect is still an unresolved issue. Researchers continue to explore innovations in CDI technology [27] (Figure 2).

### 3.2. Working Principles of CDI

#### 3.2.1. Electro-Absorption Principle

**Figure 3:**
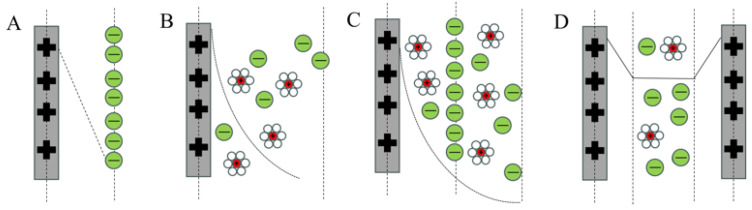
Models for elucidating the structural characteristics of an electrical double layer at a positively charged interface. (**A**) The Helmholtz model, (**B**) the Gouy−Chapman model, (**C**) the Stern model, and (**D**) the modified Donna model.

The fundamental principle underlying the electrosorption process is that upon applying a voltage across two electrodes, ions within the electrolyte are driven to move toward the electrode surfaces due to electrostatic forces. This movement aims to neutralize the charge present on the electrodes. This phenomenon is consistent with the mechanism of operation of double-electric-layer capacitors (EDL capacitors), where energy is stored by ion adsorption. The extent of double-layer electric capacitance is influenced by the electrode’s surface area. It is also affected by its capacity for charge accumulation [28]. In the 19th century, Helmholtz proposed the concept of the electric double layer (EDL) and developed a model to explain charge separation at the electrode–electrolyte interface (Figure 3A). Subsequently, Gouy and Chapman further refined Helmholtz’s model (Figure 3B). They envisaged that the ions in the electrolyte were continuously distributed in the bulk solution under the influence of thermal motion and eventually formed a diffusion layer. The Gouy–Chapman model can overestimate EDL capacitance under high-potential conditions [29]. To address this, Stern merged the Helmholtz and Gouy–Chapman models, creating a new EDL model. This model includes a compact Stern layer and a diffusion layer (Figure 3C) [30]. The compact layer encompasses the inner Helmholtz plane (IHP), which contains specifically adsorbed ions, and the outer Helmholtz plane (OHP), where counterions move across this region. The Gouy–Chapman–Stern model becomes inapplicable when diffusion layers overlap significantly within micro-pores and the Debye length surpasses the pore radius. To address this, the improved Donnan theory was introduced to describe fully overlapping EDLs, assuming a uniform electrostatic potential within the micropores, as illustrated in Figure 3D [31]. This theoretical framework suggests that salt ions occupy the complete micropore volume instead of being restricted to the pore surfaces. It is meticulously designed for this specific application. Thus, it facilitates the prediction of equilibrium salt adsorption and charge distribution within microporous carbon matrices [15].

#### 3.2.2. Advanced Characterization Techniques for Understanding Electrosorption

In the charging and discharging process, the adsorption and desorption processes of charged ions are accompanied by the complex changes in the physical and chemical properties of the electrode materials. Conventional ex situ methodologies, including nuclear magnetic resonance (NMR), infrared (IR), and Raman spectroscopy, yield crucial insights into interfacial interactions, crystalline structures, and additional pertinent properties of the electrode materials. Nonetheless, these techniques are constrained in their capacity to furnish operando data during the charge and discharge cycles. In the past decade, substantial advancements have been achieved in the realm of in situ characterization methods, aimed at enhancing the comprehension of ion electrosorption mechanisms within electrode materials. These innovations encompass in situ NMR, in situ electrochemical quartz crystal microbalance (EQCM), in situ small-angle X-ray scattering (SAXS), in situ IR, and in situ Raman spectroscopy [32]. While these complex technical approaches are primarily utilized in super capacitor research, they also possess significant application potential in CDI-related studies.

**Figure 4:**
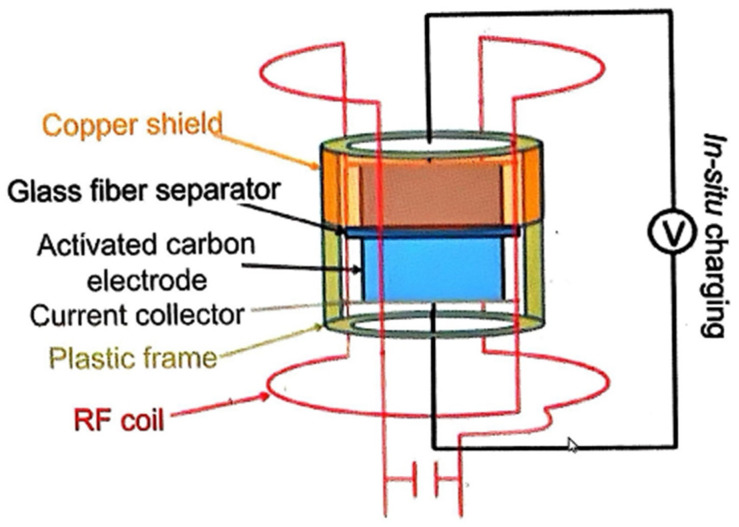
Sketch of the experimental setup for in situ NMR.

**Figure 5:**
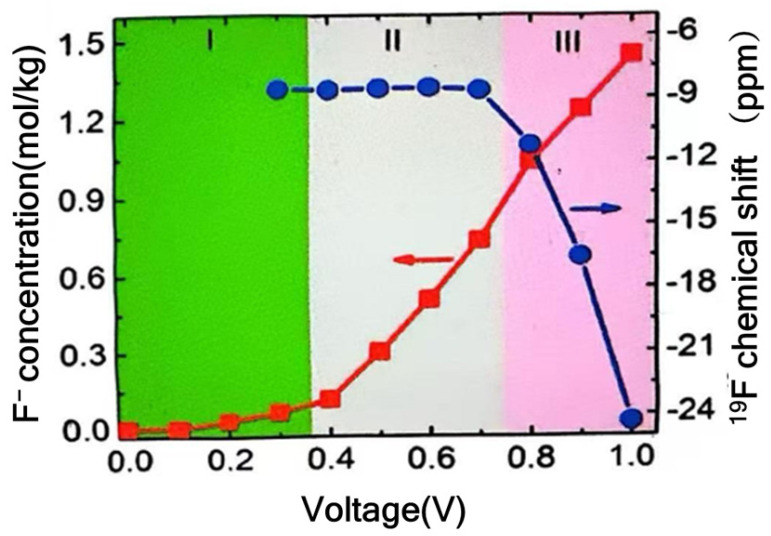
Chemical shift and intensity of F^−^ of NaF aqueous solution in nanopores.

In Situ NMR: NMR spectroscopy is utilized to examine and quantify the ion environments within porous carbon materials. The fundamental principle of nuclear magnetic resonance (NMR) technique involves exciting the nuclei of a sample under application of a strong magnetic field, followed by the measurement of the resulting nuclear resonance frequency changes to determine the physical properties of the sample. These frequencies are then converted into NMR signals. Each specific frequency offers valuable information regarding the electronic structure, dynamic behavior, and chemical surroundings of the atoms that constitute the electrode material. For example, the dynamic encapsulation of ions within a nanokernel via target electrolyte ions can be characterized by the reduction in resonance frequency, which is attributed to the shielding effect caused by the off-bound electron distribution on the carbon surface [33]. For the investigation of the charge storage mechanism in activated carbons, a typical in situ NMR scheme was employed, using a sodium fluoride aqueous solution as the electrolyte medium (in Figure 4). Figure 5 displays the chemical shift and intensity of F^−^ ions, as derived from the NMR spectra. These details provide insights into the behavior of fluoride ions under the conditions studied [34]. The data show that anions (F^−^) undergo partial dehydration as the applied voltage climbs above 0.7 V, which results in a pronounced shift in the chemical signature (represented by the blue line). When the charging voltage surpasses 0.4 V, F^−^ ions start to penetrate the pores, successfully overcoming the pre-existing energy barrier. Additionally, a substantial energy barrier was observed for ions attempting to access nanopores narrower than their hydrated ion size.

**Figure 6:**
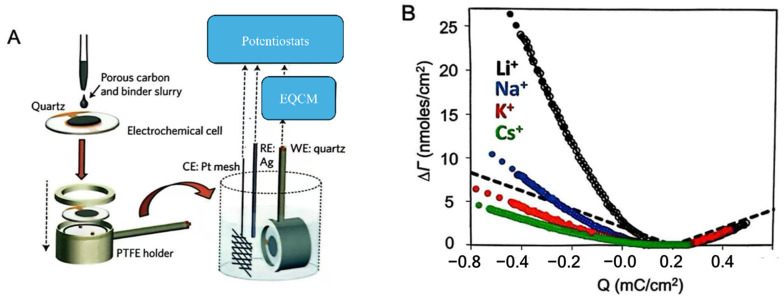
In situ EQCM cases for ion storage in nanopore carbons. (**A**) Sketch of the experimental setup for in situ EQCM. Reproduced with permission. (**B**) Variation in the adsorption of ions with respect to the accumulated charge per unit area in aqueous electrolytes with various alkali cations. Reproduced with permission.

In Situ EQCM: In situ electrochemical quartz crystal microbalance (EQCM) is a powerful technique for monitoring mass changes at the electrode–electrolyte interface. The system integrates an electrochemical cell with a quartz crystal microbalance. A thin piezoelectric quartz crystal is mounted between two metallic electrodes, enabling the generation of an alternating electric field across the crystal and inducing resonant vibrations. In this setup, the electrode in contact with the electrolyte acts as the working electrode, while a reference electrode and a counter electrode complete the electrochemical assembly. During charge–discharge processes, reactions occurring at the electrode-electrolyte interface cause shifts in the quartz crystal’s resonance frequency. These frequency changes can be mathematically correlated to mass variations via the Sauerbrey equation [35]. Consequently, EQCM provides a quantitative tool to explore the behavior of electro-adsorbed ions and solvent molecules within porous carbon structures. The in situ EQCM setup’s configuration is illustrated in Figure 6A [36]. In 2009, Levi et al. were among the first to utilize EQCM for exploring adsorption processes in porous carbon materials [37]. The ion concentration variations with charge accumulation in the porous carbon electrodes, as observed in Figure 6B, can be attributed to the polarization processes of different alkali metal cations in aqueous solutions [38]. The data show that Li^+^, Na^+^, K^+^, and Cs^+^ ions, when adsorbed, are typically accompanied by 2.0, 0.8, 0.1, and 0.0 water molecules, respectively. This observation highlights a significant reduction in solvated water molecules as ions are confined within nanopores. To investigate the correlation between ion size and pore size, researchers employed the EQCM method, incorporating a pure ionic liquid electrolyte (EMI^+^TFSI^−^) alongside carbide-derived carbons that provide a precise adjustment of pore dimensions [39]. Two specific pore sizes, 1 nm and 0.65 nm, were chosen for the study. The data reveal that in carbon materials featuring 1 nm pores, similar in size to the cations and anions, only cations engage in charge balancing under negative polarization conditions. When subjected to positive polarization, the mechanism shifts; at low charge densities, ion exchange between anions and cations is prevalent. Conversely, at high charge densities, the primary activity involves the adsorption of counterions, specifically anions. This trend holds true even when a solvent (EMI^+^TFSI^−^ in acetonitrile) is present. Nonetheless, when EMI^+^TFSI^−^ is solvated in acetonitrile, ions experience desolvation upon entering the carbon nanopores. Within 1 nm pores, cations are associated with 3–4 solvent molecules, whereas in the bulk electrolyte, this number is around eight. When the pore diameter decreases from 1 nm to 0.65 nm, the solvation numbers for cations drop from 3–4 to 1–2. These findings reveal that ions undergo partial desolvation upon entering smaller pores, with the effect intensifying in smaller pores.

**Figure 7:**
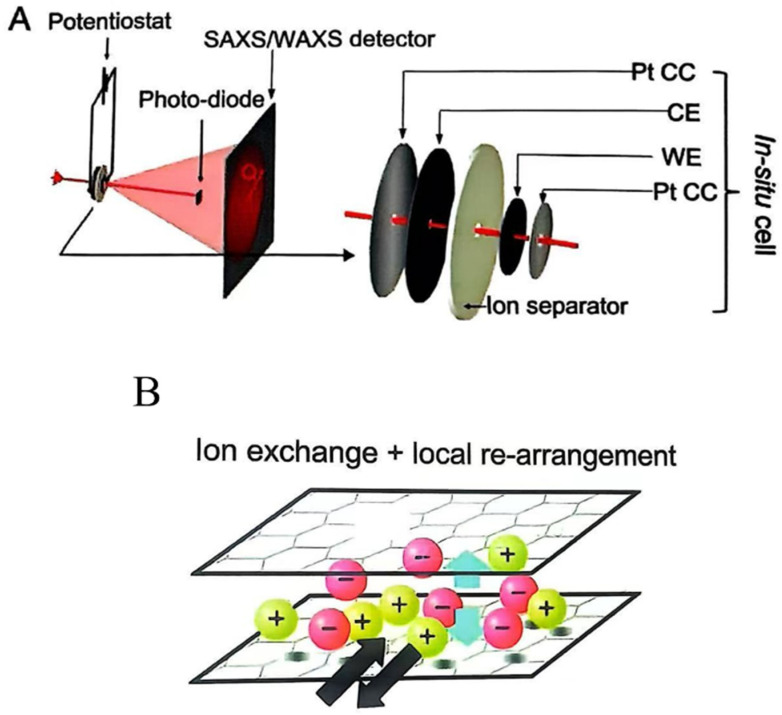
(**A**) Sketch of the experimental setup for in situ SAXS. (**B**) Sketch of ion exchange and local rearrangement when applied a voltage to nanopores. Adapted with permission. Copyright 2015, The Royal Society of Chemistry.

In Situ SAXS: Small-angle X-ray scattering (SAXS) is a potent tool for observing ion movement and concentration changes within nanopores. It functions by measuring how X-rays scatter elastically when passing through materials, specifically capturing the scattering at minimal angles. As shown in Figure 7A, the basic SAXS arrangement involves directing a monochromatic X-ray beam onto the electrodes. While the majority of X-rays pass straight through without interacting, those that scatter offer critical insights into the material’s properties. A small proportion of the X-ray photons encounters electrons within the sample, resulting in elastic scattering and a minor directional change. This scattering event is detected by a flat panel situated on the side of the sample opposite to the X-ray source, where the emerging scattering pattern is recorded [40]. Preal and their team employed this method to systematically characterize the ion structures and concentration profiles of polyanode carbon electrodes during polarization processes in various aqueous solutions, particularly in NaCl, KCl, and CsCl systems [41]. When applying a scanning voltage, a diagram illustrating the scattering intensity in relation to the scattering vector modulus (Q) can be produced. This diagram distinguishes between two zones, namely Q-A, applicable when Q is less than 5 nm^−^¹ and impacted by scattering contrast, and Q-B, relevant for Q values above 5 nm^−^¹ and directly related to ion concentration [42]. During cycling, when the voltage transitions from positive to negative, ion substitution typically occurs, leading to observed intensity changes in the CsCl electrolyte. The scattering contrast is influenced by the electron count of the ions. In the case of the KCl electrolyte, the intensity should theoretically remain constant, since both K^+^ and Cl^−^ ions possess 18 electrons. For NaCl, a reversal of the scattering signal could be expected because Na^+^ has fewer electrons than Cl^−^. Despite this, similar qualitative intensity variations were noted in both KCl and NaCl electrolytes, mirroring the changes observed in CsCl. This unexpected behavior in KCl and NaCl probably arises from solvent dynamics, particularly the exchange or condensation of water molecules. The strong hydration shells around K^+^ and Na^+^ may affect the effective electron density, potentially contributing to this deviation. By utilizing the radius of gyration (Rg) obtained from a Guinier analysis of SAXS data and the average electron density (ρel) of the electrolyte measured through X-ray transmission, a model was created to link SAXS intensity changes with applied voltage. This model illustrates the evolution of the local electrolyte environment near micropore boundaries. At low charge densities, ion substitution predominantly dictates the charge storage mechanism. Conversely, at high charge densities, counterion adsorption emerges as a crucial aspect of the charging process, alongside local ion reorganization within the pores (Figure 7B). Counterions gather near the pore walls, forming a dense layer of ions and solvent molecules [41]. A partial desolvation was observed in mixed micro- and mesoporous carbons using in situ X-ray small-angle scattering (SAXS) analysis, which was combined with Monte Carlo simulations to model the structural changes. Similar findings were observed when using in situ NMR and EQCM techniques, as SAXS provided critical insights into ion mobility and charge retention processes within the carbon’s nanoporous structure.

**Figure 8:**
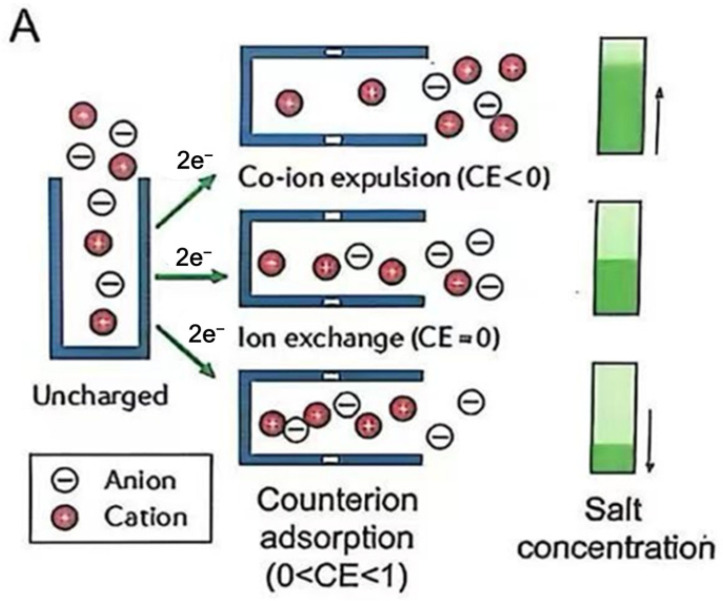

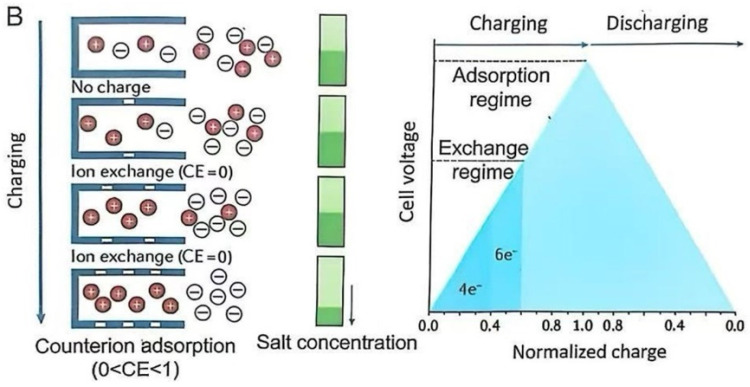
Ion electrosorption mechanism of CDI. (**A**) Charge compensation mechanisms; (**B**) ion electrosorption in nanopore under low molar strengths. Reproduced with permission. Copyright 2020, Springer Nature Limited.

In Situ IR and Raman Spectroscopy Techniques: Other cutting-edge characterization approaches, such as in situ IR and Raman spectroscopy, have been established to offer greater clarity on the mechanisms responsible for capacitive energy storage [43]. These techniques enable the tracking of ion concentration changes within porous networks. To illustrate, when carbon nanofibers undergo charging from low to high voltages, anions notably outnumber cations. Several research efforts have applied in situ Raman spectroscopy to probe the movement of ions within microporous carbons, observing that the entry and departure of ions cause discernible shifts in Raman intensity [44]. Ma et al. investigated the influence of larger metallic ions’ hydration diameter on the mobility of smaller ions, demonstrating a significant deceleration in their motion [45]. The evolution of in situ experimental techniques has played a key role in advancing our fundamental knowledge of ion behavior and charge storage within electric double layers (EDLs). Several key conclusions can be drawn regarding the behavior of ions when they electro-adsorb into nanopores. Charge Compensation Mechanisms: Figure 8A illustrates that charge adjustment is not solely managed by the adsorption of counterions. The advancement of experimental techniques in situ has been vital for deepening our understanding of ion trapping and charge storage dynamics within electric double layers (EDLs). As the energy levels increase, the charge storage mechanism shifts: counterions are forced out of the pores, followed by the adsorption of counterions, adhering to an ion-exchange pattern as illustrated in Figure 8B. For capacitive deionization (CDI), which focuses on removing salt ions from water, the expulsion of co-ions and ion exchanges do not effectively reduce the electrolyte’s ion concentration. Rather, these processes result in charge consumption, leading to low charge efficiency [46]. This points out a key difference between CDI and supercapacitors. Ideally, in a CDI system, every bit of applied charge would be used for counterion adsorption, leading to 100% charge efficiency. The differences in electrolyte concentration regimes when used for CDI applications and supercapacitor systems suggest that the charge storage mechanisms may differ, motivating further research into their underlying principles. Ions encounter energy barriers upon trying to enter nanopores, which must be overcome for them to penetrate. Additionally, ion desolvation occurs as ions enter nanopores, with the extent of desolvation increasing as the pore size decreases.

#### 3.2.3. Faraday’s Principle of Ion Removal

**Figure 9:**
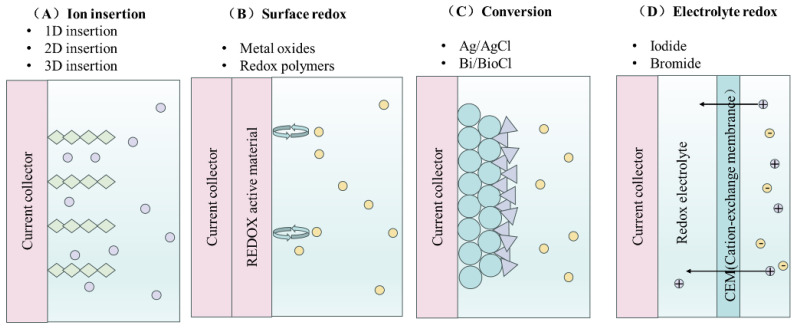
Faradaic ion capture mechanisms. (**A**) Ion insertion; (**B**) surface redox; (**C**) Faradaic conversion reaction; and (**D**) charge compensation with redox electrolyte.

Faraday reaction-induced deionization involves ions captured during charge transfer, including insertion, surface redox, transformation reactions, charge compensation of redox-active electrolytes, and changes in electrode material structure. Insertion reactions introduce a guest substance into a host structure without altering it and are typically reversible [47]. As illustrated in Figure 9A, the insertion mechanism varies with the host material’s structure. One-dimensional materials, like tunnel-structured manganese oxides, facilitate ion diffusion and storage through tunnels [48]. The interlayer spaces of two-dimensional materials, including transition metal disulfides (TMDs) and MXenes, accommodate the insertion of host ions [49]. Three-dimensional materials, including Prussian blue analogs and NASICON-like materials, feature open frameworks that provide three-dimensional ion transport channels [50].

The electrochemical current significantly depends on the potential characteristics of the inserted material. Layered materials with non-specific sites exhibit pseudo-capacitive features, while materials with specific insertion sites show a battery-like mechanism [51]. Surface redox reactions in certain electrode materials result in high pseudo-capacitance, as illustrated in Figure 9B [52]. Transition metal oxides, including MnO_x_, Co_3_O_4_, and CeO_2_, display pseudo-capacitive traits that are advantageous for desalination [53]. The low conductivity and high internal resistance of transition metal oxides (TMOs) can be enhanced by integrating them with carbon, leading to improved electrochemical performance in electrode materials [54]. Conductive polymers like polypyrrole, polyindole, and polyaniline, which have redox activity, are also effective CDI electrode materials, as they facilitate ion adsorption and desorption through oxidation and reduction states. Electrode materials facilitate new compound formation via conversion reactions. The materials commonly employed in CDI include Ag/AgCl and Bi/BiOCl, as illustrated in Figure 9C [46]. In Ag/AgCl, the oxidation reaction involves Cl^−^ reacting with Ag^+^ to create AgCl, while reduction causes Cl^−^ to be released back into the solution [55]. This technique employs Faraday materials for ion removal. The charge compensation of redox-active ions in the electrolyte aids in extracting ions from the solution, as shown in Figure 9D [56]. Redox-active ions like iodide or bromide can alter their oxidation state by gaining or losing electrons at the solid/liquid interface. Upon electrode activation, trivalent iodide ions are oxidized to monovalent iodide ions (I^3−^−2e^−^↔I^−^), achieving charge equilibrium in the redox electrolyte and attracting two cations. In the reverse reaction (I^−^ reduced to I^3−^), these cations are subsequently released into the solution [57].

#### 3.2.4. Advanced Characterization Techniques for Understanding Faradaic Storage

X-ray diffraction (XRD) serves to determine material crystal structures by subjecting samples to collimated X-rays and recording the reflected beam’s intensity. The in situ XRD method can observe changes in crystal structures throughout charge–discharge operations. Shi and his team were among the first to explore in situ XRD applications in capacitive deionization (CDI), examining a specially designed cell featuring Prussian blue (PB) adorned with polyaniline. They noted that during desalination, specific PB diffraction peaks, such as (200), (220), (400), and (420), migrate to lower angles and revert to their starting points after electrode regeneration. The sodium ion movement within the PB structure is driven by reversible insertion and extraction, thereby influencing the expansion or contraction of lattice parameters [58]. In a WS_2_/rGO electrode, a similar observation was made as the XRD profiles of WS_2_ showed a gradual shift toward lower angles. This indicates continuous sodium ion intercalation into the WS_2_ framework, accompanied by an increase in the lattice parameters [59].

In situ Raman spectroscopy provides a strong approach to recognizing the vibrational behaviors of bonds in Faradaic substances during ongoing charge and discharge events [60]. The Raman setup for CDI can be designed for direct adaptation from battery/supercapacitor research [61]. According to previous points, Raman spectroscopy is a tool for monitoring bond vibrations. MoS_2_ displays two characteristic Raman peaks, E_2g_ and A_1g_, reflecting the in-plane and out-of-plane vibrational modes of its Mo and S atoms, respectively [62].

Moreover, the A_1g_ peak shows sensitivity to the interlayer electron density. During Faradaic reactions, the binding of electrons increases the interlayer electron density of MoS_2_, leading to a decrease in the A_1g_ peak intensity and an increase in the E_2g_/A_1g_ ratio. With a decrease in potential from 0 to −0.5 V and from 0.5 to 0 V, the E_2g_/A_1g_ ratio decreases. Conversely, it increases when the potential is raised from −0.5 V to 0.5 V. This demonstrates that sodium ions participate in a reversible intercalation reaction within the MoS_2_ framework [63]. Recent studies have highlighted the significant impact of ion insertion on the structural integrity of MoS_2_ layers, a phenomenon attributed to the intercalated ions’ larger size relative to the existing interlayer spacing. Notably, the insertion of chloride ions at the positive electrode exhibits distinct thermodynamic stability compared to sodium insertion at the negative electrode. Additionally, the energy requirement for chloride intercalation at the anode is lower than that of sodium at the cathode, which can be attributed to the size difference between the inserted ions and the interlayer spacing. This finding underscores the importance of ion size in determining the stability and energy efficiency of MoS_2_-based electrode materials. In aqueous environments, ions are commonly observed as hydrated forms rather than bare ions, thereby increasing their effective radii. This research represents the first study to examine the influence of ion size on insertion and removal processes within CDI systems [64]. Recent studies have demonstrated that diverse electrolytes can deliver unique charge storage mechanisms even when employing identical Faradaic materials. Notably, the functionality of these materials is significantly influenced by the specific environments created by the electrolytes. These environments not only affect how charges are stored but also determine whether the system behaves more like a capacitor or a battery under varying conditions [65]. Therefore, selecting the appropriate electrolyte, especially aqueous solutions, requires meticulous attention. As more Faradaic materials are increasingly applied in capacitive deionization (CDI) applications, it is imperative to thoroughly examine the ion capture mechanisms for each device. Such distinctions necessitate a tailored approach, diverging from the conventional methodologies employed in battery-related studies.

## 4. Performance Indicators and Electrode Materials

The next section will focus on performance metrics for evaluating CDI and the electrode materials used in CDI configurations [66]. To ensure accurate performance evaluation, suitable metrics are crucial for comparing various CDI systems. A spectrum of CDI performance metrics, including desalination capacity, charging efficiency, the de-salination rate, cycling stability, and water recovery, are analyzed to enhance electrode stability and electrochemical performance, ultimately improving efficiency in desalination and purification processes.

Electrode materials are crucial for CDI and are classified into capacitive (non-Faraday) and pseudo-capacitive (Faraday) types based on ion storage mechanisms. CDI electrodes often benefit from the use of capacitive materials, which are characterized by their high surface area, excellent conductivity, and superior water stability. These materials are particularly advantageous in applications requiring robust performance under challenging conditions, such as in water treatment systems. Their ability to maintain consistent functionality in adverse environments underscores their suitability for advanced electrochemical applications [67]. Activated carbon, carbon nanotubes, graphene, and MOF-derived carbon are typical capacitive materials used in CDI. Recently, Faraday electrode materials have gained prominence, presenting new opportunities for CDI. This chapter summarizes CDI performance metrics and the selectivity mechanisms of Faraday materials, comparing their selectivity with traditional capacitive materials [68].

### 4.1. Performance Indicators

Key performance indicators for capacitive desalination (CDI) devices, known as desalting capacity (SAC), represent the efficiency of removing dissolved salts from brine solutions. SAC achieves its maximum value when equilibrium salt adsorption is attained. In CDI experiments, equilibrium is established by maintaining a stable cell voltage or current and controlling the inlet water flow rate until the cell attains full charge. This ensures optimal desalination performance through electrokinetic properties. Under steady-state conditions, the adsorption process ceases, and the electrical conductivity of the cell lysate becomes time-invariant, consequently exhibiting a stabilized state. SAC can be divided into gravimetric adsorption capacity (GAC) and volumetric adsorption capacity (VAC). GAC measures the mass of salt removed per unit mass of electrode, while VAC quantifies the volume of salt per unit electrode volume. The second aspect primarily investigates the energy efficiency and energy dissipation characteristics of the CDI system during the charging and discharging processes. Energy requirements are directly influenced by the initial brine concentration and the total salt to be removed. This highlights the importance of optimizing operational parameters and module design to enhance desalination performance while minimizing energy consumption. Charge efficiency (Λ) is key—a higher Λ means lower energy use. Λ is the ratio of adsorbed salt to charge, calculated by dividing the charge in the electrodes by Faraday’s constant to obtain moles. Among the critical metrics of the CDI process, the salt adsorption rate (SAR) serves as a key indicator, calculated by dividing SAC by the charging or total cycling time, and it is typically evaluated on a per mg/g-min basis. The Kim-Yoon diagram, a CDI Ragone plot, illustrates the relationship between SAR and SAC, facilitating the determination of optimal cell performance and operating conditions. CDI performance metrics are essential for the ongoing growth of CDI research. SAC measures the desalination capability of electrode materials, while the charge efficiency (Λ) indicates system efficiency. These metrics help compare various CDI systems and other desalination technologies [69].

### 4.2. Electrode Materials

#### 4.2.1. Capacitor Materials

Capacitive materials primarily consist of carbon-based substances, which are classified into unmodified and modified types. Unmodified carbon materials depend on ion physical properties (hydration radius, valence, electronegativity) and weak interactions with the carbon electrode for selective adsorption [70]. A 2001 study by Eliad found that monovalent ions, having a smaller hydration radius, exhibit higher selectivity in porous carbon electrodes compared to divalent ions [71]. Studies revealed that the pore size distribution in activated carbon cloth electrodes significantly affects their selectivity. Electrodes with high microporosity tend to adsorb ions with smaller hydration radii. Zafra et al. report that Cl^−^ and NO_3_^−^ ions, having smaller sizes, are more easily adsorbed compared to H_2_PO_4_^−^/HPO_4_^2−^ ions, which is attributed to the average pore size (approximately 0.855 nm) of the activated carbon material [72]. The valence state of ions influences their selectivity in CDI, with higher valence ions being more easily adsorbed due to stronger electrostatic forces. Gao et al. found that charge repulsion at carbon nanotube electrodes also enhances the selectivity of higher valence ions [73]. Xing et al. used a one-dimensional EDL model to show that in a mixed Cl^−^ and ClO_4_^−^ salt solution, ClO_4_^−^ is selectively adsorbed despite its lower concentration due to its higher diffusion rate within the carbon pores (9 × 10^−10^ m^2^/s for ClO_4_^−^, 1 × 10^−10^ m^2^/s for Cl^−^); the carbon electrode still showed selectivity for ClO_4_^−^. Chemical modification can enhance ion selectivity in the CDI process [74]. For instance, Gao et al. used acid treatment to create an amino-modified activated carbon electrode that selectively adsorbed SO_4_^2−^.

In addition to the distinct physicochemical properties of competing ions and target ions, the introduction of functional groups onto carbon electrodes through chemical modification can also enhance the ion selectivity of capacitive deionization (CDI). Oyarzun et al. functionalized the surface of carbon-based electrodes through the use of cetyltrimethylammonium bromide (CTAB) and sodium dodecylbenzene sulfonate (SDBS), which are well-known surface-active agents. This modification process was attributed to the specific properties of these additives, resulting in a modified electrode with a nitrate (NO_3_^−^) adsorption rate 7.7 times higher than that of chloride (Cl^−^) [75]. This selectivity was unaffected by variations in the NO_3_^−^/Cl^−^ concentration. Furthermore, CHEN et al. prepared amino-modified activated carbon electrodes through acid treatment, achieving a selective adsorption of sulfate (SO_4_^2−^) [76]. The authors attributed this enhanced selectivity to stronger hydrogen bonding between the amino groups and SO_4_^2−^, increasing the selectivity for SO_4_^2−^ from 0.64 to 1.27–1.61. MIAO et al. developed a CDI electrode based on nitric acid-treated activated carbon to effectively remove phosphate ions (PO_4_^3−^) from wastewater contaminated with chloride ions (Cl^−^), phosphate ions (PO_4_^3−^), and sulfate ions (SO_4_^2−^). This innovative electrode was designed to selectively address phosphate removal while maintaining selectivity against other ions present in the wastewater [77]. The selective performance of phosphate ions (PO_4_^3−^) was enhanced by a factor of six when compared with untreated activated carbon, and this improvement was accompanied by an increase in selectivity efficiency as the surface content of carboxyl functional groups on the electrode increased.

Liu et al. developed an EDTA-functionalized graphene electrode for selective heavy metal ion recovery through strong coordination [78]. Ji et al. proposed a method based on the Lewis acid–base theory [79]. The study by Deng et al. used density functional theory (DFT) simulations to investigate how acidic (carboxyl) and basic (amino) functional groups affect the adsorption of Cl^−^, NO_3_^−^, H_2_PO_4_^−^, and SO_4_^2−^ anions [80]. They found that carboxyl groups promoted the selective separation of H_2_PO_4_^−^, while amino groups enhanced the selective adsorption of both H_2_PO_4_^−^ and SO_4_^2−^.

**Figure 10:**
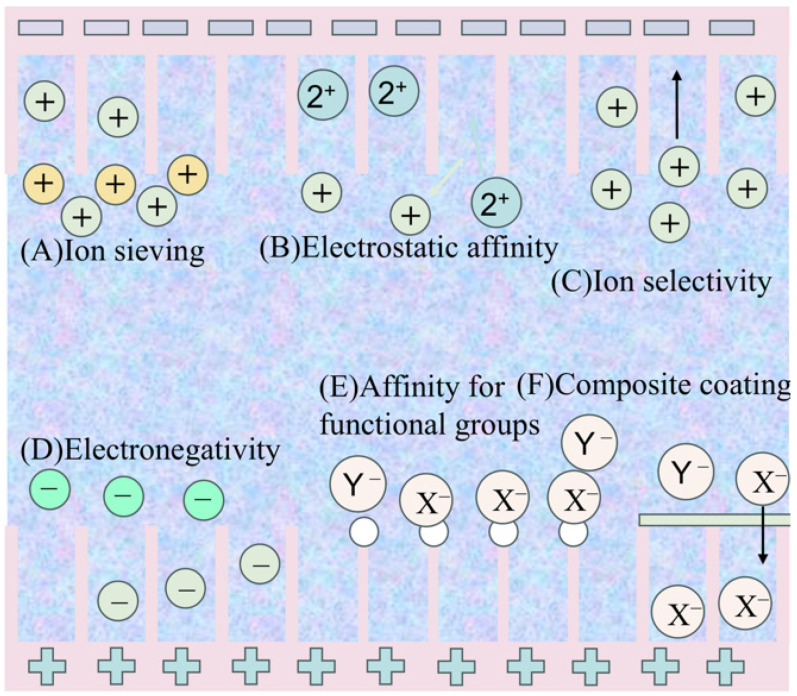
Principle of CDI carbon-based capacitive materials to achieve selective separation.

Zhang et al. investigated the selective removal of phosphate in capacitive deionization (CDI) using a composite material of carbon nanotubes modified with terephthalic acid (ZnZr-COOH/CNT) as the anode [81]. The presence of impurity ions such as Cl^−^, NO_3_^−^, and SO_4_^2−^ had minimal impact on phosphate adsorption, and the effluent phosphate concentration remained below the national first-level discharge standard (0.5 mg/L).The selective adsorption of phosphate was enhanced through the formation of complexes between Zr, Zn, and phosphate, as well as hydrogen bonding between the hydroxyl groups of phosphate and the carboxyl groups of the electrode material. Zuo et al. synthesized a hybrid Co-MOF@rGO nanomaterial by the in situ growth of nanocrystals on the surface of reduced graphene oxide (rGO), which demonstrated a selective removal and conversion of Cr(VI) [82]. The material demonstrated enhanced Cr(VI) removal efficiency by integrating collective adsorption, electrocatalytic reduction, and desorption mechanisms. Through synergistic interactions, the material effectively eliminated Cr(VI) ions from aqueous solutions and transformed them into a safer, lower oxidation state. This process, driven by the material’s unique structural properties, significantly improved the overall performance of Cr(VI) removal in various applications. The Co-MOF in the composite provided high affinity for CrO_4_^2−^, while the rGO contributed the necessary electrical conductivity. In conclusion, different chemical modifications can confer specific selective characteristics to carbon electrodes (Figure 10).

#### 4.2.2. Pseudo-Capacitor Materials

**Figure 11:**
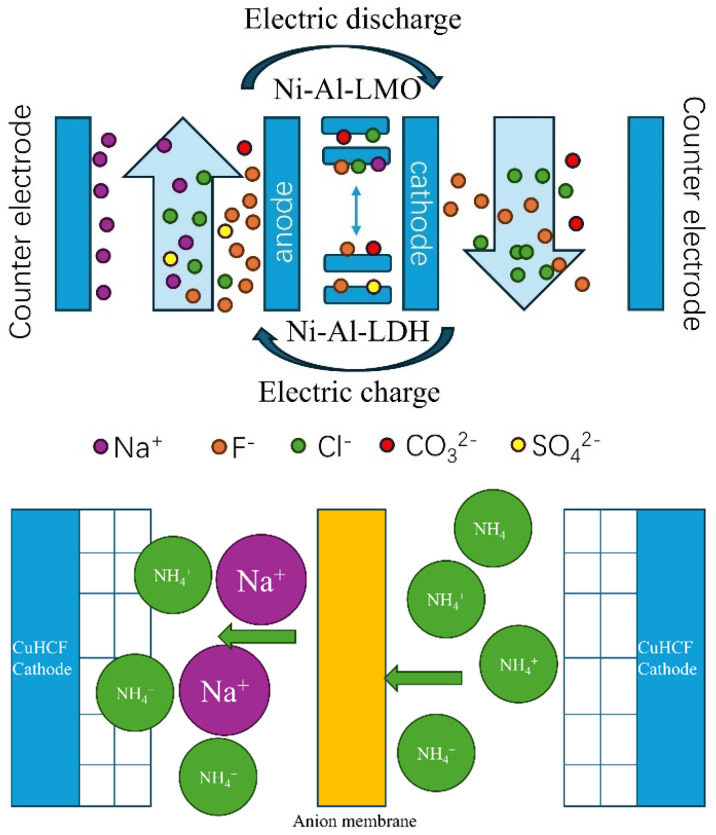
Mechanism of selective removal of different types of pseudo-capacitor materials.

Pseudo-capacitive materials, such as inorganic conductors and redox-active substances, are employed as selective adsorption electrodes within the context of CDI. This is primarily due to their exceptional ions’ adsorption capacity and their strong affinity for binding with the surrounding medium. Examples like LDHs (layered double hydroxides), LMOs (layered metal oxides), and PBAs (Prussian blue analogs) can store ions within their crystal structures, allowing for high-capacity storage without needing a large surface area [83]. Based on the layered metal oxide (Ni-Al-LMO) electrode, BAI et al. selectively adsorbed fluoride ions from drinking water. The high electronegativity of fluoride ions and their paired ligand effect with hydroxyl–aluminum clusters led to an adsorption capacity of approximately 50 mg/g for F^−^, which exhibited superior adsorption performance compared to chlorine ions (fourfold increase) [84]. Kim et al. used sodium–manganese oxide (Na_0.44_MnO_2_) to selectively adsorb Na^+^ from various cationic solutions, with a selectivity ratio of 13 for Na^+^ over K^+^ at the same ionic concentration and a selectivity range of 6 to 8 for Na^+^ over Mg^2+^ [85]. CuHCF is a Prussian blue analog. Kim et al. applied a low voltage (0.1 to 0.3 V) to a two-electrode electrochemical cell to achieve the selective removal of NH^4+^. The selectivity of the CuHCF electrode for Na^+^ is over fourfold, and approximately 80% of NH^4+^ can be removed. Tsai et al. used nickel hexacyanoferrate (NiHCF) to treat municipal wastewater, finding that cations with smaller hydration radii and lower hydration energies were preferentially absorbed into the NiHCF lattice, and the order of cation selection by the NiHCF electrode was NH_4_^+^ > K^+^ > Na^+^ > Ca^2+^ > Mg^2+^. DFT simulations aid in understanding electrode selectivity for specific ions [86]. Jiang et al. calculated the binding energy and volume change in Li^+^, Na^+^, and K^+^ in the CuHCF lattice, discovering that K^+^ embedding results in the greatest decrease in binding energy and the smallest lattice volume change [87]. This insight supports the development of new insertion materials with enhanced selectivity. The limited electrical conductivity and easy dissolution of intercalation materials hinder their long-term use [88]. Shi et al. studied how various synthesis methods, including chelating agents and different precursors, affect the Na^+^ adsorption and life cycle of CuHCF electrodes. They found that smaller precursor crystals (under 35 nm) and lower structural water content improved the Na^+^ removal rate from 40.4 mAh/g to 53.2 mAh/g but decreased long-term cycling stability (maintaining 20% to 55% selectivity after 100 cycles). In contrast, the formation of a Cu(II)/Cu(I) redox pair using a chelator as a precursor improved the stability of the cycling performance of the Fe(III)/Fe(II) redox pair for CDI (79.4% selectivity retained after 100 cycles). In summary, electrode materials are crucial for the CDI selective separation process, but most studies lack long-term stability data. Issues like carbon oxidation, intercalation structure decomposition, and membrane contamination by heavy metals affect stability. Enhancing long-term electrode stability is an urgent challenge. Pan et al. suggested enhancing HCDI cycling performance by using polyimide with redox activity as the cathode instead of traditional inorganic compounds and by employing nickel hexacyanoferrate/graphene oxide as the cathode material to operate at low voltage and prevent oxidation on the activated carbon anode [89] (Figure 11).

## 5. Ion-Selective Membrane

The previous sections have extensively discussed methods and performance improvements for achieving ion selectivity through electrode design. However, in capacitive deionization (CDI), the application of membranes is equally critical. Numerous studies have demonstrated the advantages of ion-exchange membranes (IEMs) in preventing co-ion expulsion, reducing anode oxidation, and enhancing desalination in multi-chamber systems [90]. IEMs not only act as barriers for specific ions but also significantly improve ion selectivity. Therefore, this section focuses on summarizing the approaches to achieving ion selectivity through the use of membranes.

### 5.1. Cation-Exchange Membrane

Cation-exchange membranes (CEMs) typically possess a polymer backbone that is characterized by a range of negatively charged functional groups, including carboxylate, sulfonate, and phenolate ions. These functional groups enable the selective passage of cations while excluding anions [91]. Additionally, some layered metal oxide (LMO)-based materials demonstrate high affinity for particular single-valent ions, such as the monovalent cation-selective membranes CSO (Selémion) and CIMS (Neosepta), which exhibit strong ion adsorption capabilities. The CSO membrane contains a positively charged layer, which due to charge repulsion, more effectively repels divalent cations compared to monovalent cations. The CIMS membrane, on the other hand, possesses a highly crosslinked (bulk) structure that permits the passage of monovalent cations with smaller hydrated radii while blocking divalent cations with larger hydrated radii [92]. Choi et al. observed a monovalent cation selectivity (R) of 1.8 for Na^+^/Ca^2+^ when using the CIMS membrane, achieving a concentrated Ca^2+^ solution through the selective removal of Na^+^. Furthermore, the selectivity reached its maximum under conditions of higher cell voltage, pH, and lower total dissolved solid concentration. This selective behavior is attributed to the presence of the proton-exchange membrane (PEM), which induces stronger charge repulsion against higher-valency ions.

### 5.2. Anion-Exchange Membrane

Similarly to the use of CEMs, anion-exchange membranes (AEMs) are also commonly employed in studies on ion selectivity in membrane capacitive deionization (MCDI). Research has shown that AEMs can promote the desorption of certain anions, with MCDI exhibiting lower removal rates for sulfate compared to chloride and nitrate [93]. A novel approach to enhancing MCDI selectivity involves modifying ion-exchange membranes (IEMs) with polyelectrolytes, which can act as selective layers through electrostatic interactions. Singh et al. combined a nickel hexacyanoferrate (NiHCF) electrode with an AEM coated with poly(diallyldimethylammonium chloride) (PDADMAC) and poly(sodium 4-styrenesulfonate) (PSS), achieving a simultaneous selective separation of monovalent and divalent cations, as well as anions [94]. The modified membrane demonstrated a superior adsorption of divalent anions over monovalent anions, while the NiHCF electrode showed a better adsorption of monovalent cations over divalent cations. The separation factor (β) for Cl^−^ over SO_4_^2−^ ranged between 7 and 14, while the average separation factor (β) for Na^+^ over Mg^2+^ was 17, reflecting the ion selectivity of the modified membrane and electrode, respectively. This selectivity remained consistent even at low concentrations of monovalent ions.

### 5.3. Ion-Exchange Resin Coating

Similarly to ion-exchange membranes (IEMs), the development of ion-exchange resin coatings applied to electrode interfaces is another effective method to enhance the selectivity of capacitive deionization (CDI). Due to the affinity of polystyrene sulfonate (PSS) for divalent ions, Nnorom et al. adjusted the water absorption, ion-exchange capacity, and permeation selectivity of PSS by varying its crosslinking density with the electrode, enabling its use as an ion-selective layer in membrane capacitive deionization (MCDI) processes. This significantly improved the selective removal performance for Ca^2+^, with a selectivity coefficient of approximately eight for Ca^2+^/Na^+^ [95]. Kim et al. selectively removed Ca^2+^ in the presence of Na^+^ by coating-activated carbon electrodes with a calcium-selective nanocomposite (CSN) resin layer [96]. When the feed solution had a Ca^2+^/Na^+^ ratio of 1:1, the CSN-coated electrode exhibited a 50.7% higher adsorption capacity for Ca^2+^ and a 48% reduction in Na^+^ adsorption compared to uncoated electrodes.

Yeo et al. investigated the selectivity of CDI systems for nitrate by coating the anode with a nitrate-selective ion-exchange resin and compared it with systems using AEMs [97]. Cation-exchange resins, which are strong base anion exchangers, exhibit excellent selectivity for nitrate ions due to their long alkyl chains. In MCDI systems, the selectivity coefficients for NO_3_^−^/Cl^−^ and SO_4_^2−^/Cl^−^ were approximately 2 and 1.3, respectively, consistent with trends reported in other studies [98]. After coating the electrode with the resin, the selectivity for NO_3_^−^/Cl^−^ and NO_3_^−^/SO_4_^2−^ increased to 3.7 and 1.3, respectively. Kim et al. obtained similar results using nitrate-selective resins (NO_3_^−^/Cl^−^ ≈ 3.2) [99]. Although nitrate-selective resins have consistently demonstrated excellent NO_3_^−^ selectivity in the literature, recent studies have found that the desorption efficiency of NO_3_^−^ is relatively low compared to competing anions, requiring longer times to achieve complete desorption [100].

Similar studies to those of Yeo et al., such as those conducted by Zuo et al., have also explored comparable aspects in their research, such as modified carbon electrodes with a quaternary ammonium-functionalized polyvinyl alcohol (QPVA) binder layer containing submicron-sized sulfate-selective ion-exchange resin particles [101]. These particles facilitated the preferential migration of SO_4_^2−^ to adsorption sites on the carbon surface, achieving the selective adsorption of SO_4_^2−^. Even at a Cl^−^:SO_4_^2−^ molar ratio as high as 20:1, the system exhibited significant selectivity. The SO_4_^2−^/Cl^−^ selectivity increased from 1.37 at the beginning of adsorption to 2.24 at equilibrium, whereas uncoated electrodes showed higher selectivity for chloride over sulfate (Si/j = 2.2). Sun et al. reported similar findings [102], demonstrating that coating-activated carbon electrodes with selective ion-exchange resins could reverse the SO_4_^2−^/Cl^−^ selectivity to 2.4. Even when the Cl^−^ concentration increased by 100-fold, the resin-coated carbon maintained a sulfate selectivity of 1.9.

## 6. Problems Faced and Solutions

Despite significant advancements in CDI research over the past 20 years, there is still considerable potential for improvement in selective separation applications. The focus has been primarily on developing electrode and membrane materials, while the optimization and design of CDI systems have been largely overlooked and need more research attention. Evaluating CDI systems’ performance in selective separation is essential [103]. Additionally, in-depth experimental validation and real-life application studies are needed. Ongoing research in these areas will advance CDI technology. Below are the potential problems and challenges in this exploration.

### 6.1. Water Oxidation

Water oxidation during CDI can occur on the positively charged electrode, leading to oxygen formation and cation removal. However, this reaction competes with ion adsorption, lowering the charge efficiency of ion electrosorption [104]. The alteration in chemistry at the electrode–electrolyte interface can lead to electrode corrosion over repeated cycles. To mitigate water oxidation side reactions, appropriate voltage control and positive potential management are essential, though this approach may compromise ion removal efficiency [105]. The authors suggest using electrode materials with high oxidation overpotentials to minimize water oxidation even under a current. Carbon-based materials, including graphite and activated carbon, are widely utilized in cathode degradation prevention technologies (CDI) due to their inherent low reactivity characteristics. Additionally, other carbon-based materials within this category are also commonly employed in these systems for similar reasons. Adjusting parameters like voltage, water flow rate, temperature, and pH can enhance ion adsorption and minimize water oxidation. For instance, reducing voltage decreases water oxidation, and protective coatings on electrodes can prevent direct contact with water molecules, further reducing oxidation [106]. Researchers have investigated the use of catalytic coatings to selectively promote water oxidation kinetics under a wider potential window. In conclusion, water oxidation and its effects remain a key factor hindering the design of CDI cells to achieve optimized salt adsorption capacity and charge efficiency while maintaining stable electrode performance.

### 6.2. Dissolved Oxygen Reduction

Dissolved oxygen reduction occurs at the negative electrode of CDI cells during the adsorption phase, where oxygen molecules gain electrons. This side reaction can impact electrode performance and ion removal efficiency in CDI. Specifically, the reduction in dissolved oxygen to hydrogen molecules may occur on the surface of the negatively polarized electrode, while anions may compete for adsorption at the electrode surface. This may lead to a decrease in the electrosorption efficiency of the anions, reducing the effectiveness of ion removal [107]. Furthermore, the dissolved oxygen reduction reaction alters the chemical composition of the electrode–electrolyte interface, which may contribute to corrosion and degradation of the electrode performance. This alteration in chemical composition could also result in modifications to the interface properties, potentially exacerbating corrosion issues and further reducing the system’s efficiency [108]. In order to reduce the effects of dissolved oxygen reduction, a number of measures can be taken. Firstly, the relationship between desalination efficiency and oxygen reduction can be balanced by precisely regulating the operating voltages to avoid unnecessary high-voltage operation. Secondly, some electrode materials with high overpotential for oxygen reduction reactions are selected, which means that these materials are not prone to oxygen reduction reactions even if a certain voltage exists. For example, some noble metals and their alloys, or modified carbon materials, can be candidates [109]. Finally, modifying the surface of the electrode by chemical or physical methods can change its catalytic activity and thus inhibit the occurrence of the oxygen reduction reaction. For example, a layer of nanoparticles or other materials can be deposited on the electrode surface to make it more favorable for the adsorption of target ions while reducing the possibility of oxygen reduction. The concentration of dissolved oxygen can also be reduced by passing an inert gas, such as nitrogen, through the electrolyte [110]. In addition to this, oxidizing agents can be used to promote the oxidation of dissolved oxygen, thereby reducing the likelihood of its involvement in the electrode reaction. In conclusion, dissolved oxygen reduction is an important factor affecting the performance of CDI, and its effect needs to be reduced by proper operation and design to improve the ion removal efficiency and stability of CDI.

### 6.3. Electrode Fouling

Electrode fouling constitutes a critical concern that significantly influences the performance of conventional direct injection (CDI) systems. During operation, specific ions from the water, along with other elements, may irreversibly adhere to the carbon micropores. Additionally, the accumulation of organic compounds and minerals on the porous electrodes may exacerbate fouling issues [111]. Fouling clogs the pores and hides the active adsorption sites, thereby impeding the migration of ions into the micropores and reducing the electrosorption capacity during cycling. In addition, fouling can make the battery more resistive by hindering ion migration within the electrode [112]. In order to avoid electrode fouling, several methods can be used to reduce the risk of electrode surface fouling by firstly removing most of the suspended and soluble minerals from the raw water by filtration, softening, or other pre-treatment techniques. Secondly, selecting or developing some electrode materials with better anti-fouling performance. For example, some new materials such as titanium alloys, coated electrodes, etc. [113], because of their surface properties, are not conducive to mineral attachment and can reduce the scaling phenomenon. Specifically, the surface of titanium alloys usually naturally forms a thin and dense titanium oxide (TiO_2_) film, which not only improves the corrosion resistance of the material but also reduces the adsorption of other substances on its surface. Especially when a specific oxidation treatment is used, the oxide layer can be made more uniform and stable, thus enhancing the anti-scaling effect, while some coating materials can prevent the attachment of specific ions or molecules and others have special wettability, which can change the way the liquid medium interacts with the solid surface, such as super-hydrophobic coatings, because they allow almost no droplets to stay, greatly reducing the likelihood of mineral deposition. In addition, an appropriate amount of anti-fouling agent can be added to the water, such as a dispersant or scale inhibitor; these chemicals can prevent the formation of solid deposits of minerals on the electrode. Through the above measures, the impact of electrode scaling on the CDI system can be effectively reduced to ensure the long-term stable operation of the system [114].

## 7. Outlook

Electrochemical deionization (ECDI) is an innovative water treatment technology that efficiently recovers valuable elements from industrial wastewater. It combines an electric field with specialized electrodes to selectively extract ions, offering environmental and cost benefits over traditional adsorption methods. Research in recent years has focused on enhancing the elemental selective extraction efficacy of ECDI systems, especially in the application of valuable elemental resources.

This paper analyzes the evolution of CDI technology, its core principles, and recent advancements in selective ion removal and recovery. It highlights that through the detailed study of ion storage mechanisms, electrode material optimization, solvent effects, and reactor design, ECDI has achieved significant progress in seawater desalination and target element extraction. However, challenges persist with cycling durability, adsorption/desorption efficiency, charge utilization efficiency, and the ion selectivity of electrode materials, which are key areas for future technological advancements. Another key point is that CDI can selectively separate target ions in multi-component solutions, not just desalinate, depending on the electrode or membrane used.

Electrode selectivity relies on ion sieving, hydration energy/ratio, affinity to functional groups, electronegativity, and site-specific reactions. Ion-exchange membranes or resins are crucial for membrane ion selectivity. Future research should focus on designing electrode materials and developing membranes to enhance target ion selectivity. Machine learning can enhance CDI technology development, particularly in capacitors, by accelerating material discovery and design. Algorithms can predict properties like capacitance, conductivity, and stability, aiding researchers in identifying superior capacitor materials. Its application in capacitors is focused on several aspects as follows:

1. Material discovery and design: The discovery of new materials can be accelerated through machine learning algorithms. For example, training models to predict the properties of different material combinations, such as capacitance values, conductivity, stability, etc., can help researchers find new materials with excellent properties. Machine learning can also be used to optimize the microstructure of materials, such as the pore distribution of porous carbon materials, to achieve higher specific capacitance.
2. Manufacturing optimization: In the manufacturing process of capacitors, machine learning can be used to optimize process parameters such as temperature, pressure, reaction time, etc., to improve product quality and consistency. Three-dimensional printing technology combined with machine learning can enable a customized design of the internal structure to make them more suitable for specific application scenarios.
3. Performance prediction and modeling: Based on historical experimental data, the machine learning model can predict the performance of capacitors under different conditions, such as temperature changes, the charge/discharge rate, and other factors on their life and performance. It can also help engineers to consider the impact of these factors at the design stage.
4. Fault detection and diagnosis: Machine learning can be used to monitor the working status of capacitors, provide early warning of potential failures, extend service life, and reduce maintenance costs.
5. Intelligent control of capacitors: In some application scenarios, such as power systems or battery management systems for electric vehicles, machine learning algorithms can be used to optimize the charging and discharging strategies of capacitors for optimal energy management. For example, researchers at Georgia Tech used supercomputers and machine learning techniques to accelerate the analysis of electronic materials, which helped them find ways to improve capacitors faster. Meanwhile, research has shown that a combination of 3D printing technology and machine learning can produce carbon micro-lattices with customized properties that can be used as energy storage elements for supercapacitors. These applications demonstrate the potential of machine learning to enhance the development, production, and application of capacitors, helping to advance capacitor technology.

The future of selective capacitive deionization (CDI) hinges on material innovation, system optimization, and a deeper understanding of ion-selective mechanisms. Advanced electrode materials, such as hierarchical porous structures, redox-active intercalation materials (MXenes, Prussian blue analogs), and surface-functionalized coatings, are pivotal for enhancing ion selectivity by leveraging differences in hydration radii and redox reactions. System designs like membrane-assisted CDI (MCDI) and hybrid architectures (flow-electrode CDI coupled with selective adsorbents) enable a modular design. Beyond desalination, CDI shows promise in resource recovery (Li^+^, PO_4_^3−^) and environmental remediation (Pb^2+^, As^3+^ removal). A deeper theoretical framework is needed to address multi-ion competition, dynamic selectivity, and standardized metrics, supported by advanced characterization techniques (in situ spectroscopy, DFT) and integration with circular economy practices (battery recycling). By harmonizing these advancements, CDI can evolve into a versatile platform for sustainable water purification and resource recovery, addressing both technical and application-driven challenges. Taking into account the numerous challenges involved, which span across mechanism investigations, electrode design, and cell architecture, there remains significant opportunities for the selective separation of elements within CDI technology.

In conclusion, capacitive deionization (CDI) has emerged as a transformative technology in the realm of water treatment, offering a sustainable and efficient approach to desalination and selective ion separation. Over the past decades, CDI has evolved from its initial conceptualization to advanced configurations such as membrane capacitive deionization (MCDI), flow capacitive deionization (FCDI), hybrid capacitive deionization (HCDI), and asymmetric capacitive deionization (A-CDI). These advancements have been driven by a deeper understanding of ion storage mechanisms, electrode material optimization, and reactor design, enabling CDI to address not only water scarcity but also the recovery of valuable elements from industrial wastewater.

Despite its significant progress, CDI technology still faces several challenges that hinder its widespread adoption. Key issues include the limited cycle life of electrodes, poor reversibility of adsorption/desorption processes, low charge utilization efficiency, and insufficient ion selectivity. These limitations underscore the need for continued innovation in electrode materials, membrane design, and system optimization. Furthermore, the integration of CDI with renewable energy sources and the development of energy-efficient operational strategies are critical for enhancing its sustainability and scalability.

The potential of CDI extends beyond desalination, offering promising applications in resource recovery, environmental remediation, and energy storage. The ability to selectively separate target ions from multicomponent solutions opens new avenues for the extraction of valuable metals and the removal of harmful contaminants. However, achieving these goals requires a multidisciplinary approach, combining materials science, electrochemistry, and environmental engineering.

Future research should focus on the development of advanced electrode materials with enhanced selectivity, stability, and conductivity. The exploration of novel membrane materials and configurations, as well as the optimization of operational parameters, will further improve the performance and efficiency of CDI systems. Additionally, the integration of machine learning and artificial intelligence in the design and optimization of CDI systems holds great promise for accelerating material discovery, predicting system performance, and enabling intelligent control.

In summary, CDI technology represents a promising solution to global water challenges, with the potential to contribute significantly to sustainable development. By addressing the current limitations and leveraging emerging technologies, CDI can evolve into a versatile and efficient tool for water treatment, resource recovery, and environmental protection. Continued collaboration between academia, industry, and policymakers will be essential for realizing the full potential of CDI and ensuring its successful implementation in real-world applications.

## References

[1] Wang M., Bodirsky B.L., Rijneveld R., Beier F., Bak M.P., Batool M., Droppers B., Popp A., van Vliet M.T.H., Strokal M. (2024). A triple increase in global river basins with water scarcity due to future pollution. Nat. Commun..

[2] Xu X., Eguchi M., Asakura Y., Pan L., Yamauchi Y. (2023). Metal-organic framework derivatives for promoted capacitive deionization of oxygenated saline water. Energy Environ. Sci..

[3] Sun K., Tebyetekerwa M., Wang C., Wang X., Zhang X., Zhao X.S. (2023). Electrocapacitive Deionization: Mechanisms, Electrodes, and Cell Designs. Adv. Funct. Mater..

[4] Ren Y., Nie L., Wang L., Wang X. (2023). Capacitive deionization technology and its application in heavy metal separation and recovery. China Environ. Sci..

[5] Wang L., Zhong Y., Wang H., Malyi O.I., Wang F., Zhang Y., Hong G., Tang Y. (2024). New Emerging Fast Charging Microscale Electrode Materials. Small.

[6] Lv H., Wang X., Yang Y., Liu T., Zhang L. (2023). RGO-Coated MOF-Derived In_2_Se_3_ as a High-Performance Anode for Sodium-Ion Batteries. Acta Phys.-Chim. Sin..

[7] Gong A., Liu Y., Zhao Y., Li K. (2021). Research progress on material modification and device improvement of capacitive deionization. Ind. Water Treat..

[8] Ali A., Tufa R.A., Macedonio F., Curcio E., Drioli E. (2018). Membrane technology in renewable-energy-driven desalination. Renew. Sustain. Energy Rev..

[9] Imbrogno J., Belfort G. (2016). Membrane Desalination: Where Are We, and What Can We Learn from Fundamentals?. Annu. Rev. Chem. Biomol. Eng..

[10] Deshmukh A., Boo C., Karanikola V., Lin S., Straub A.P., Tong T., Warsinger D.M., Elimelech M. (2018). Membrane distillation at the water-energy nexus: Limits, opportunities, and challenges. Energy Environ. Sci..

[11] Ghaffour N., Missimer T.M., Amy G.L. (2013). Technical review and evaluation of the economics of water desalination: Current and future challenges for better water supply sustainability. Desalination.

[12] Pan S.Y., Snyder S.W., Lin Y.J., Chiang P.C. (2018). Electrokinetic desalination of brackish water and associated challenges in the water and energy nexus. Environ. Sci.-Water Res. Technol..

[13] Sadrzadeh M., Mohammadi T. (2008). Sea water desalination using electrodialysis. Desalination.

[14] Suss M.E., Presser V. (2018). Water Desalination with Energy Storage Electrode Materials. Joule.

[15] Suss M.E., Porada S., Sun X., Biesheuvel P.M., Yoon J., Presser V. (2015). Water desalination via capacitive deionization: What is it and what can we expect from it?. Energy Environ. Sci..

[16] Remillard E.M., Shocron A.N., Rahill J., Suss M.E., Vecitis C.D. (2018). A direct comparison of flow-by and flow-through capacitive deionization. Desalination.

[17] Yu F., Yang Z., Cheng Y., Xing S., Wang Y., Ma J. (2022). A comprehensive review on flow-electrode capacitive deionization: Design, active material and environmental application. Sep. Purif. Technol..

[18] Lee J.B., Park K.K., Eum H.M., Lee C.W. (2006). Desalination of a thermal power plant wastewater by membrane capacitive deionization. Desalination.

[19] Biesheuvel P.M., van der Wal A. (2010). Membrane capacitive deionization. J. Membr. Sci..

[20] Biesheuvel P.M., Zhao R., Porada S., van der Wal A. (2011). Theory of membrane capacitive deionization including the effect of the electrode pore space. J. Colloid Interface Sci..

[21] Liu M., Xu M., Xue Y., Ni W., Huo S., Wu L., Yang Z., Yan Y.-M. (2018). Efficient capacitive deionization using natural basswood-derived, freestanding, hierarchically porous carbon electrodes. Acs Appl. Mater. Interfaces.

[22] Jeon S.I., Park H.R., Yeo J.G., Yang S., Cho C.H., Han M.H., Kim D.K. (2013). Desalination via a new membrane capacitive deionization process utilizing flow-electrodes. Energy Environ. Sci..

[23] Rommerskirchen A., Ohs B., Hepp K.A., Femmer R., Wessling M. (2018). Modeling continuous flow-electrode capacitive deionization processes with ion-exchange membranes. J. Membr. Sci..

[24] Lee J., Kim S., Kim C., Yoon J. (2014). Hybrid capacitive deionization to enhance the desalination performance of capacitive techniques. Energy Environ. Sci..

[25] Vengatesan M.R., Darawsheh I.F.F., Govindan B., Alhseinat E., Banat F. (2019). Ag-Cu bimetallic nanoparticle decorated graphene nanocomposite as an effective anode material for hybrid capacitive deionization (HCDI) system. Electrochim. Acta.

[26] Liu Q., Li X., Xiao D. (2022). Design of a novel asymmetric capacitive deionization device with high desalination performance. Environ. Sci.-Water Res. Technol..

[27] Yong T., Li Y., Lu J., Lin R., Wu J., Zuo X. (2023). Research progress of flow-electrode capacitive deionization technology. Ind. Water Treat..

[28] Lu Q., Liu P., Zhou T., Huang R., Zhang K., Hu L., Liu R., Ren Z., Wang J., Wang X. (2024). Recent progress on electro-sorption technology for lithium recovery from aqueous sources. Nano Res..

[29] Goodisman J. (1975). Lippmann equation and gouy-chapman model. J. Chim. Phys. Phys.-Chim. Biol..

[30] Oliva C., Zambrano F., Wolff D. (1985). Adsorption of univalent and divalent-cations to planar bilayer-membranes containing sulfatids or phosphatiddylserine. Biochem. Int..

[31] Kim J., Rotenberg B. (2024). Donnan equilibrium in charged slit-pores from a hybrid nonequilibrium molecular dynamics/monte carlo method with ions and solvent exchange. arXiv.

[32] Pal B., Yasin A., Kaur R., Tebyetekerwa M., Zabihi F., Yang S., Yang C.-C., Sofer Z., Jose R. (2021). Understanding electrochemical capacitors with in-situ techniques. Renew. Sustain. Energy Rev..

[33] Wang H., Forse A.C., Griffin J.M., Trease N.M., Trognko L., Taberna P.-L., Simon P., Grey C.P. (2013). In Situ NMR Spectroscopy of Supercapacitors: Insight into the Charge Storage Mechanism. J. Am. Chem. Soc..

[34] Luo Z.-X., Xing Y.-Z., Liu S., Ling Y.-C., Kleinhammes A., Wu Y. (2015). Dehydration of Ions in Voltage-Gated Carbon Nanopores Observed by in Situ NMR. J. Phys. Chem. Lett..

[35] Levi M.D., Daikhin L., Aurbach D., Presser V. (2016). Quartz Crystal Microbalance with Dissipation Monitoring (EQCM-D) for in-situ studies of electrodes for supercapacitors and batteries: A mini-review. Electrochem. Commun..

[36] Salanne M., Rotenberg B., Naoi K., Kaneko K., Taberna P.L., Grey C.P., Dunn B., Simon P. (2016). Efficient storage mechanisms for building better supercapacitors. Nat. Energy.

[37] Levi M.D., Salitra G., Levy N., Aurbach D., Maier J. (2009). Application of a quartz-crystal microbalance to measure ionic fluxes in microporous carbons for energy storage. Nat. Mater..

[38] Levi M.D., Sigalov S., Aurbach D., Daikhin L. (2013). In Situ Electrochemical Quartz Crystal Admittance Methodology for Tracking Compositional and Mechanical Changes in Porous Carbon Electrodes. J. Phys. Chem. C.

[39] Tsai W.Y., Taberna P.L., Simon P. (2014). Electrochemical Quartz Crystal Microbalance (EQCM) Study of Ion Dynamics in Nanoporous Carbons. J. Am. Chem. Soc..

[40] Li T., Senesi A.J., Lee B. (2016). Small Angle X-ray Scattering for Nanoparticle Research. Chem. Rev..

[41] Prehal C., Weingarth D., Perre E., Lechner R.T., Amenitsch H., Paris O., Presser V. (2015). Tracking the structural arrangement of ions in carbon supercapacitor nanopores using in situ small-angle X-ray scattering. Energy Environ. Sci..

[42] Gibaud A., Xue J.S., Dahn J.R. (1996). A small angle X-ray scattering study of carbons made from pyrolyzed sugar. Carbon.

[43] Chen D., Ding D., Li X., Waller G.H., Xiong X., El-Sayed M.A., Liu M. (2015). Probing the Charge Storage Mechanism of a Pseudocapacitive MnO_2_ Electrode Using *in Operando* Raman Spectroscopy. Chem. Mater..

[44] Richey F.W., Tran C., Kalra V., Elabd Y.A. (2014). Ionic Liquid Dynamics in Nanoporous Carbon Nanofibers in Supercapacitors Measured with *in Operando* Infrared Spectroelectrochernistry. J. Phys. Chem. C.

[45] Ma H., Chen H., Wu M., Chi F., Liu F., Bai J., Cheng H., Li C., Qu L. (2020). Maximization of Spatial Charge Density: An Approach to Ultrahigh Energy Density of Capacitive Charge Storage. Angew. Chem.-Int. Ed..

[46] Srimuk P., Su X., Yoon J., Aurbach D., Presser V. (2020). Charge-transfer materials for electrochemical water desalination, ion separation and the recovery of elements. Nat. Rev. Mater..

[47] Li Q., Zheng Y., Xiao D., Or T., Gao R., Li Z., Feng M., Shui L., Zhou G., Wang X. (2020). Faradaic electrodes open a new era for capacitive deionization. Adv. Sci..

[48] Post J.E. (1999). Manganese oxide minerals: Crystal structures and economic and environmental significance. Proc. Natl. Acad. Sci. USA.

[49] Smith K.C., Dmello R. (2016). Na-Ion Desalination (NID) Enabled by Na-Blocking Membranes and Symmetric Na-Intercalation: Porous-Electrode Modeling. J. Electrochem. Soc..

[50] Shin Y.-U., Lim J., Boo C., Hong S. (2021). Improving the feasibility and applicability of flow-electrode capacitive deionization (FCDI): Review of process optimization and energy efficiency. Desalination.

[51] Augustyn V., Gogotsi Y. (2017). 2D materials with nanoconfined fluids for electrochemical energy storage. Joule.

[52] Shao Y., El-Kady M.F., Sun J., Li Y., Zhang Q., Zhu M., Wang H., Dunn B., Kaner R.B. (2018). Design and mechanisms of asymmetric supercapacitors. Chem. Rev..

[53] Tang K., Hong T.Z.X., You L., Zhou K. (2019). Carbon-metal compound composite electrodes for capacitive deionization: Synthesis, development and applications. J. Mater. Chem. A.

[54] Guo X., Zhang G., Li Q., Xue H., Pang H. (2018). Non-noble metal-transition metal oxide materials for electrochemical energy storage. Energy Storage Mater..

[55] Srimuk P., Husmann S., Presser V. (2019). Low voltage operation of a silver/silver chloride battery with high desalination capacity in seawater. Rsc Adv..

[56] Lukatskaya M.R., Dunn B., Gogotsi Y. (2016). Multidimensional materials and device architectures for future hybrid energy storage. Nat. Commun..

[57] Lee J., Srimuk P., Carpier S., Choi J., Zornitta R.L., Kim C., Aslan M., Presser V. (2018). Confined redox reactions of lodide in carbon nanopores for fast and energy-efficient desalination of brackish water and seawater. ChemSusChem.

[58] Shi W.H., Liu X.Y., Deng T.Q., Huang S.Z., Ding M., Miao X.H., Zhu C.Z., Zhu Y.H., Liu W.X., Wu F.F. (2020). Enabling Superior Sodium Capture for Efficient Water Desalination by a Tubular Polyaniline Decorated with Prussian Blue Nanocrystals. Adv. Mater..

[59] Vafakhah S., Saeedikhani M., Huang S., Yan D., Leong Z.Y., Wang Y., Hou L., Guo L., Alvarado P., Yang H.Y. (2021). Tungsten disulfide-reduced GO/CNT aerogel: A tuned interlayer spacing anode for efficient water desalination. J. Mater. Chem. A.

[60] Zhao E.Y., Nie K.H., Yu X.Q., Hu Y.S., Wang F.W., Xiao J., Li H., Huang X.J. (2018). Advanced Characterization Techniques in Promoting Mechanism Understanding for Lithium-Sulfur Batteries. Adv. Funct. Mater..

[61] Jung H., Gerasopoulos K., Talin A.A., Ghodssi R. (2017). A platform for in situ Raman and stress characterizations of V_2_O_5_ cathode using MEMS device. Electrochim. Acta.

[62] Tebyetekerwa M., Zhang J., Saji S.E., Wibowo A.A., Rahman S., Truong T.N., Lu Y., Yin Z., Macdonald D., Nguyen H.T. (2021). Twist-driven wide freedom of indirect interlayer exciton emission in MoS_2_/WS_2_ heterobilayers. Cell Rep. Phys. Sci..

[63] Han J.L., Yan T.T., Shen J.J., Shi L.Y., Zhang J.P., Zhang D.S. (2019). Capacitive Deionization of Saline Water by Using MoS_2_-Graphene Hybrid Electrodes with High Volumetric Adsorption Capacity. Environ. Sci. Technol..

[64] Srimuk P., Lee J., Fleischmann S., Choudhury S., Jaeckel N., Zeiger M., Kim C., Aslan M., Presser V. (2017). Faradaic deionization of brackish and sea water via pseudocapacitive cation and anion intercalation into few-layered molybdenum disulfide. J. Mater. Chem. A.

[65] Zhang Q.N., Levi M.D., Dou Q.Y., Lu Y.L., Chai Y.G., Lei S.L., Ji H.X., Liu B., Bu X.D., Ma P.J. (2019). The Charge Storage Mechanisms of 2D Cation-Intercalated Manganese Oxide in Different Electrolytes. Adv. Energy Mater..

[66] Zhao J., Burke A.F. (2021). Review on supercapacitors: Technologies and performance evaluation. J. Energy Chem..

[67] Xu K.L., Liu J., Yan Z.X., Jin M., Xu Z.H. (2021). Synthesis and use of hollow carbon spheres for electric double-layer capacitors. New Carbon Mater..

[68] Tseng Y.F., Mofarah S.S., Zheng X., Arandiyan H., Wang Y., Abbasi R., Gao Y., Sorrell C.C., Koshy P. (2023). Engineering of micro-mesoporous two-dimensional CeO_2_-based heterojunction oxides for energy storage applications. Surf. Interfaces.

[69] Kim T., Yoon J. (2015). CDI ragone plot as a functional tool to evaluate desalination performance in capacitive deionization. RSC Adv..

[70] Fan X., Zhang H., Wei Y., Huang Y., He H., Wang Y., Meng Q., Wu W. (2023). Study of a mixed conductive layer fabricated by ion implantation and distribution Theory. Polymers.

[71] Eliad L., Salitra G., Soffer A., Aurbach D. (2001). Ion sieving effects in the electrical double layer of porous carbon electrodes: Estimating effective ion size in electrolytic solutions. J. Phys. Chem. B.

[72] Macias C., Lavela P., Rasines G., Zafra M.C., Tirado J.L., Ania C.O. (2014). Improved electro-assisted removal of phosphates and nitrates using mesoporous carbon aerogels with controlled porosity. J. Appl. Electrochem..

[73] Gao K., Lin X., Yu W., Cheng X., Zhang S., Li S., Zhang Z. (2022). Few-layered ReS_2_@CNTs as a high-performance cathode for aluminum-ion batteries. Adv. Mater. Interfaces.

[74] Jiao S., Zheng J., Li Q., Li X., Engelhard M.H., Cao R., Zhang J.G., Xu W. (2018). Behavior of lithium metal anodes under various capacity utilization and high current density in lithium metal batteries. Joule.

[75] Oyarzun D.I., Hemmatifar A., Palko J.W., Stadermann M., Santiago J.G. (2018). Ion selectivity in capacitive deionization with functionalized electrode: Theory and experimental validation. Water Res. X.

[76] Chen X., Deng W., Miao L., Gao M., Ao T., Chen W., Ueyama T., Dai Q. (2023). Selectivity adsorption of sulfate by amino-modified activated carbon during capacitive deionization. Environ. Technol..

[77] Miao L., Deng W., Chen X., Gao M., Chen W., Ao T. (2021). Selective adsorption of phosphate by carboxyl-modified activated carbon electrodes for capacitive deionization. Water Sci. Technol..

[78] Liu P., Yan T., Zhang J., Shi L., Zhang D. (2017). Separation and recovery of heavy metal ions and salt ions from wastewater by 3D graphene-based asymmetric electrodes via capacitive deionization. J. Mater. Chem. A.

[79] Ji Q., An X., Liu H., Guo L., Qu J. (2015). Electric Double-Layer Effects Induce Separation of Aqueous Metal Ions. Acs Nano.

[80] Deng W., Chen Y., Wang Z., Chen X., Gao M., Chen F., Chen W., Ao T. (2022). Regulation, quantification and application of the effect of functional groups on anion selectivity in capacitive deionization. Water Res..

[81] Zhang H., Wang Q., Zhang J., Chen G., Wang Z., Wu Z. (2022). Development of novel ZnZr-COOH/CNT composite electrode for selectively removing phosphate by capacitive deionization. Chem. Eng. J..

[82] Zuo K., Huang X., Liu X., Garcia E.M.G., Kim J., Jain A., Chen L., Liang P., Zepeda A., Verduzco R. (2020). A Hybrid Metal-Organic Framework-Reduced Graphene Oxide Nanomaterial for Selective Removal of Chromate from Water in an Electrochemical Process. Environ. Sci. Technol..

[83] Dong T., Zhang W., Zheng W. (2024). Origin of pseudocapacitance and achieving bulk pseudocapacitance. J. Chin. Ceram. Soc..

[84] Feng J., Bai H., Xue Y., Zhang R., Zhu P., Bu D., Dan Z., Li W., Lu X. (2020). Recycling of iron and aluminum from drinking water treatment sludge for synthesis of a magnetic composite material (ALCS-Fe-Al) to remove fluoride from drinking water. Groundw. Sustain. Dev..

[85] Kim H., Kim D.J., Seo D.-H., Yeom M.S., Kang K., Kim D.K., Jung Y. (2012). Ab initio study of the sodium intercalation and intermediate phases in Na_0.44_MnO_2_ for sodium-ion battery. Chem. Mater..

[86] Tsai S.W., Cuong D.V., Hou C.H. (2022). Selective capture of ammonium ions from municipal wastewater treatment plant effluent with a nickel hexacyanoferrate electrode. Water Res..

[87] Jiang P., Shao H., Chen L., Feng J., Liu Z. (2017). Ion-selective copper hexacyanoferrate with an open-framework structure enables high-voltage aqueous mixed-ion batteries. J. Mater. Chem. A.

[88] Song D., Wang S., Liu R., Jiang J., Jiang Y., Huang S., Li W., Chen Z., Zhao B. (2019). Ultra-small SnO_2_ nanoparticles decorated on three-dimensional nitrogen-doped graphene aerogel for high-performance bind-free anode material. Appl. Surf. Sci..

[89] Ding Z., Xu X., Li Y., Wang K., Lu T., Pan L. (2019). Significantly improved stability of hybrid capacitive deionization using nickel hexacyanoferrate/reduced graphene oxide cathode at low voltage operation. Desalination.

[90] Zornitta R.L., Ruotolo L.A.M. (2018). Corrigendum to “Simultaneous analysis of electrosorption capacity and kinetics for CDI desalination using different electrode configurations” [Chem. Eng. J. 332 (2018) 33–41]. Chem. Eng. J..

[91] Fuoco A., Khdhayyer M.R., Attfield M.P., Esposito E., Jansen J.C., Budd P.M. (2017). Synthesis and Transport Properties of Novel MOF/PIM-1/MOF Sandwich Membranes for Gas Separation. Membranes.

[92] Luo T., Abdu S., Wessling M. (2018). Selectivity of ion exchange membranes: A review. J. Membr. Sci..

[93] Omosebi A., Gao X., Landon J., Liu K. (2014). Asymmetric Electrode Configuration for Enhanced Membrane Capacitive Deionization. Acs Appl. Mater. Interfaces.

[94] Singh K., Sahin S., Gamaethiralalage J.G., Zornitta R.L., de Smet L.C.P.M. (2022). Simultaneous, monovalent ion selectivity with polyelectrolyte multilayers and intercalation electrodes in capacitive deionization. Chem. Eng. J..

[95] Nnorom N.C., Rogers T., Jain A., Alazmi A., Elias W.C., DuChanois R.M., Flores K., Gardea-Torresdey J.L., Cokar M., Elimelech M. (2022). Sulfonated polymer coating enhances selective removal of calcium in membrane capacitive deionization. J. Membr. Sci..

[96] Kim J., Jain A., Zuo K., Verduzco R., Walker S., Elimelech M., Zhang Z., Zhang X., Li Q. (2019). Removal of calcium ions from water by selective electrosorption using target-ion specific nanocomposite electrode. Water Res..

[97] Yeo J.-H., Choi J.-H. (2013). Enhancement of nitrate removal from a solution of mixed nitrate, chloride and sulfate ions using a nitrate-selective carbon electrode. Desalination.

[98] Uzun H.I., Debik E. (2019). Economical approach to nitrate removal via membrane capacitive deionization. Sep. Purif. Technol..

[99] Kim Y.-J., Choi J.-H. (2012). Selective removal of nitrate ion using a novel composite carbon electrode in capacitive deionization. Water Res..

[100] Gan L., Wu Y., Song H., Zhang S., Lu C., Yang S., Wang Z., Jiang B., Wang C., Li A. (2019). Selective removal of nitrate ion using a novel activated carbon composite carbon electrode in capacitive deionization. Sep. Purif. Technol..

[101] Zuo K., Kim J., Jain A., Wang T., Verduzco R., Long M., Li Q. (2018). Novel Composite Electrodes for Selective Removal of Sulfate by the Capacitive Deionization Process. Environ. Sci. Technol..

[102] Sun Z., Chai L., Liu M., Shu Y., Li Q., Wang Y., Qiu D. (2018). Effect of the electronegativity on the electrosorption selectivity of anions during capacitive deionization. Chemosphere.

[103] Askari M., Rajabzadeh S., Tijing L., Shon H.K. (2024). Advances in capacitive deionization (CDI) systems for nutrient recovery from wastewater: Paving the path towards a circular economy. Desalination.

[104] Zhang C., He D., Ma J., Tang W., Waite T.D. (2018). Faradaic reactions in capacitive deionization (CDI)—Problems and possibilities: A review. Water Res..

[105] Balaji Wright A., Wu J., Shocron A.N., Dana A.G., Suss M., Mani A. (2024). Understanding degradation of capacitive deionization cells: Full-cell simulations with anode corrosion. Desalination.

[106] Cohen I., Avraham E., Bouhadana Y., Soffer A., Aurbach D. (2015). The effect of the flow-regime, reversal of polarization, and oxygen on the long term stability in capacitive de-ionization processes. Electrochim. Acta.

[107] Shapira B., Avraham E., Aurbach D. (2016). Side reactions in capacitive deionization (CDI) processes: The role of oxygen reduction. Electrochim. Acta.

[108] Lee J.-H., Bae W.-S., Choi J.-H. (2010). Electrode reactions and adsorption/desorption performance related to the applied potential in a capacitive deionization process. Desalination.

[109] Srimuk P., Ries L., Zeiger M., Fleischmann S., Jaeckel N., Tolosa A., Kruener B., Aslan M., Presser V. (2016). High performance stability of titania decorated carbon for desalination with capacitive deionization in oxygenated water. Rsc Adv..

[110] Celebanska A., Opallo M. (2016). Layer-by-layer gold-ceramic nanoparticulate electrodes for electrocatalysis. Chemelectrochem.

[111] Mossad M., Zou L. (2013). Study of fouling and scaling in capacitive deionisation by using dissolved organic and inorganic salts. J. Hazard. Mater..

[112] Wang T., Liang H., Bai L., Liu B., Zhu X., Wang J., Xing J., Ren N., Li G. (2020). Desalination performance and fouling mechanism of capacitive deionization: Effects of natural organic matter. J. Electrochem. Soc..

[113] Kim H., Choi Y., Lee S., Lee K.-B., Jung K.-W., Choi J.-W. (2021). Pretreatment for capacitive deionization: Feasibility tests using activated filter media and granule activated carbon filtration. J. Ind. Eng. Chem..

[114] Xu Q., He R., Li Y. (2023). Problems and Mistakes for Electron Transfer Mechanism in Z-Scheme Photocatalytic System. Acta Phys.-Chim. Sin..

